# Aspirin Dosing Frequency and Dose Influence Thromboxane Suppression Despite Uniform Inhibition of Arachidonic Acid-induced Platelet Aggregation

**DOI:** 10.1055/a-2885-2306

**Published:** 2026-06-12

**Authors:** John W. Eikelboom, Sonia S. Anand, Debi A. Sloane, Jack Hirsh, Huyen A. Tran, Qilong Yi, Jeffrey I. Weitz

**Affiliations:** 1 33493 Population Health Research InstituteHamiltonOntarioCanada; 2 139264 The Thrombosis and Atherosclerosis Research InstituteHamiltonOntarioCanada; 3 Department of Medicine 3710 McMaster UniversityHamiltonOntarioCanada; 4 Department of Clinical Haematology 5392 Alfred HealthMelbourneVictoriaAustralia; 5 Department of Clinical Haematology 549290 The Australian Centre for Blood Diseases, Monash UniversityMelbourneVictoriaAustralia; 6 Department of Epidemiology and Community Medicine 177403 School of Epidemiology and Public Health, University of OttawaOttawaOntarioCanada; 7 Department of Biochemistry and Biomedical Sciences 3710 McMaster UniversityHamiltonOntarioCanada; 8 Department of Medical Sciences 3710 McMaster UniversityHamiltonOntarioCanada

**Keywords:** aspirin, thromboxane, platelet function, cardiovascular disease

## Abstract

**Background:**

Incomplete thromboxane suppression with 81 mg aspirin daily is common and predicts higher vascular risk. We compared the effects of aspirin in controls and patients with cardiovascular disease and tested strategies to improve thromboxane suppression.

**Methods:**

Two-phase randomized, open-label pharmacodynamic study. In Phase 1, controls and patients received enteric-coated (EC) aspirin 81 mg daily for 3 weeks. In Phase 2, the best responders within each cohort were randomized to EC aspirin 81 mg daily, EC aspirin 81 mg alternate daily, or soluble aspirin 81 mg daily, and the poorest responders to EC aspirin 81 mg daily, 325 mg daily, or 162 mg twice daily. Where results with EC and soluble aspirin 81 mg daily were similar, these arms were pooled for comparisons of daily versus alternate daily dosing. Outcome measures were light transmission aggregation in response to arachidonic acid, adenosine diphosphate, and collagen; serum thromboxane B2 (sTXB2); and urinary 11-dehydro-thromboxane B2 (uTXB2; creatinine-normalized).

**Results:**

We enrolled 136 controls and 115 patients. Among controls, EC aspirin 81 mg daily suppressed platelet aggregation and reduced sTXB2 from 238.4 to 2.7 ng/mL and uTXB2 from 112.0 to 39.0 ng/mmol creatinine (all *p* < 0.0001). After 3 weeks, aggregation and serum thromboxane were similar in controls and patients but uTXB2 remained higher in patients (46.5 vs. 39.0, *p* = 0.004). In Phase 2, different aspirin formulations, doses, and dose frequencies did not materially affect platelet aggregation. In the best responders, EC and soluble aspirin had similar effects on thromboxane, but sTXB2 levels were higher with alternate daily versus daily dosing (pooled EC and soluble) in both controls (8.1 vs. 2.2 ng/mL, *p* = 0.005) and patients (5.2 vs. 1.7 ng/mL, *p* = 0.048). In the poorest responders, EC aspirin 162 mg twice daily produced the greatest suppression of both sTXB2 and uTXB2, with intermediate suppression with EC aspirin 325 mg daily and the least with EC aspirin 81 mg daily (all trend *P* values <0.05).

**Conclusion:**

Daily low-dose aspirin suppresses aggregation and serum thromboxane, but urinary thromboxane metabolites are less completely suppressed in patients with established cardiovascular disease. Alternate daily dosing attenuates thromboxane suppression, whereas divided dosing improves suppression in the poorest responders.

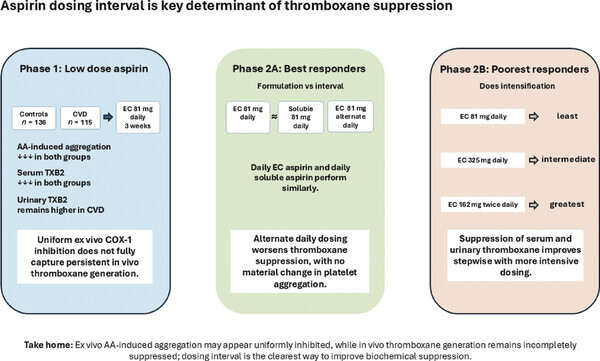

## Introduction

Aspirin reduces the risk of non-fatal and fatal cardiovascular events in a broad range of high-risk patients. 1 Its antiplatelet effect is mediated primarily by irreversible acetylation of platelet cyclooxygenase-1 (COX-1), thereby preventing the generation of thromboxane A2 (TXA2), a potent platelet agonist. 2 Low-dose aspirin produces near-complete inhibition of platelet COX-1, consistent with evidence from randomized trials that doses of 75 to 150 mg/day are as effective as higher doses for long-term secondary cardiovascular prevention. 1 3 In contrast to the observed inhibition of ex-vivo platelet COX-1, biochemical measures of thromboxane generation in vivo (such as urinary 11-dehydro-thromboxane B2) are variably suppressed by aspirin, and less complete suppression predicts subsequent cardiovascular events. 4 5 Potential explanations for the incomplete suppression of thromboxane production include non-adherence, reduced absorption (including enteric-coated [EC] formulations), 6 7 increased platelet turnover leading to substantial recovery of thromboxane production in the 24-hour dosing interval, and thromboxane generation from non-platelet sources (including COX-2–dependent inflammatory pathways). 5 8

This study aimed to examine factors that influence incomplete thromboxane suppression by aspirin. In our study we administered EC aspirin 81 mg daily to controls without cardiovascular disease and to patients with known cardiovascular disease to identify the best and poorest responders. Subsequently, participants were randomized based on their responses to various interventions designed to mimic non-adherence (alternate daily dosing), modified absorption (soluble formulation), and increased dose and dosing frequency.

## Methods

### Study Design

This was a two-phase, randomized, open-label, pharmacodynamic study involving parallel groups of community controls and patients with established cardiovascular disease. The protocol included a 3-week screening phase (phase 1) followed by a 3-week experimental phase (phase 2) comprising Part A (best responders) and Part B (poorest responders). The protocol was approved by the Hamilton Integrated Research Ethics Board, and all participants provided written informed consent.

### Participants

Controls were eligible if they were 30 years or older and had no known cardiovascular disease. During the study, the inclusion criteria for controls were relaxed to allow enrollment of individuals with cardiovascular risk factors, thereby facilitating recruitment. Patients were eligible if they were 30 years or older and had known cardiovascular disease defined as prior coronary or peripheral artery disease, or ischemic stroke. Key exclusions included known aspirin intolerance, concomitant anticoagulant therapy, antiplatelet therapy other than aspirin, active bleeding or high bleeding risk, recent (within 3 months) acute thrombosis or major intercurrent illness, and regular use of non-selective NSAIDs/COX-2 inhibitors or acid-suppressive therapy (H2 blockers or proton pump inhibitors) when discontinuation was not feasible.

### Interventions

Phase 1: All participants received EC aspirin 81 mg daily for 3 weeks.

Phase 2: Participants were categorized as best or poorest responders based on prespecified functional and biochemical criteria measured at the end of phase 1. Best responders were defined as having arachidonic acid–induced aggregation <20% and urinary 11-dehydro-thromboxane B2 (uTXB2) below the 75th percentile of the control distribution; in controls, best responders additionally required ≥95% inhibition of serum thromboxane B2 (sTXB2) from baseline (this could not be evaluated in patients because they were already treated with aspirin at baseline). The poorest responders were those who did not meet one or more of these criteria.

In Part A, best responders were randomized to: (1) EC aspirin 81 mg daily; (2) EC aspirin 81 mg alternate daily; or (3) soluble aspirin 81 mg daily, for 3 weeks. In Part B, the poorest responders were randomized to: EC aspirin (1) 81 mg daily; (2) 325 mg daily; or (3) 162 mg twice daily, for 3 weeks.

### Sample Collection and Laboratory Testing

Blood and urine specimens were obtained at baseline in controls prior to starting aspirin (not in patients because most were already taking aspirin prior to study entry), and in both patients and controls at the end of phase 1 and at the end of phase 2. Visits were scheduled in the morning, before the next scheduled aspirin dose. Venous blood (approximately 30 mL) was collected with minimal stasis by standard phlebotomy into 3.2% sodium citrate Vacutainer tubes (9:1 vol/vol; Becton, Dickinson and Company [BD], Franklin Lakes, NJ, USA) for platelet aggregation testing and serum Vacutainer tubes (BD) for serum thromboxane measurements. A first-morning urine specimen was collected at each visit, aliquoted, and stored at −80°C for batch analysis.

Platelet aggregation was assessed by light transmission aggregometry in platelet-rich plasma and performed immediately after blood collection in the Hemostasis Reference Laboratory (Hamilton Regional Laboratory Medicine Program, McMaster University Medical Centre). Platelet-rich plasma was prepared by centrifugation of citrated blood at room temperature (200g for 10 minutes) and platelet-poor plasma by further centrifugation (2000g for 15 minutes). Aggregation was measured using a four-channel optical aggregometer (Chrono-Log Corp., Havertown, PA, USA) at 37°C with constant stirring and reported as the maximal percent change in light transmission relative to platelet-poor plasma. Agonists (final concentrations) were arachidonic acid 1.0 mmol/L, ADP 5.0 µmol/L, and collagen 1.25 µg/mL. Light transmission aggregometry was performed in accordance with published consensus recommendations. 9

Serum thromboxane B2 (sTXB2) was measured as a biochemical index of platelet COX-1 activity. Whole blood collected into plain serum tubes was incubated at 37°C for 60 minutes to permit thromboxane generation during clotting, centrifuged, and serum was aliquoted and stored at −80°C until batch analysis. Urinary 11-dehydro-thromboxane B2 (uTXB2) was measured as an index of in-vivo thromboxane generation. sTXB2 and uTXB2 were quantified in duplicate using commercial enzyme immunoassay kits (Cayman Chemical, Ann Arbor, MI, USA) according to the manufacturer's instructions. Absorbance was measured on a standard microplate reader, and concentrations were derived from a four-parameter logistic standard curve. uTXB2 concentrations were normalized to urinary creatinine (Jaffe method). Pre-analytical handling and frozen storage followed standard recommendations. 10

### Statistical Analysis

Baseline characteristics are presented as means (standard deviations) or counts (percentages) and compared between patients and controls using Student's t-test and chi-square test, for continuous and categorical variables, respectively. Laboratory outcomes are presented as *n* , median, and interquartile range (IQR). For phase 2 analyses, EC aspirin 81 mg once daily and soluble aspirin 81 mg once daily were pooled as a daily 81 mg group when results were similar (for comparisons with alternate daily dosing). Within-control comparisons (baseline vs. end of phase 1) used paired non-parametric tests (Wilcoxon signed-rank test); between-group comparisons used non-parametric methods (Wilcoxon rank sum test). Two-sided *P* values <0.05 were considered statistically significant. Analyses were performed in SAS version 9.4 (SAS Institute Inc., Cary, NC, USA).

## Results

Overall, 251 participants were enrolled (136 controls and 115 patients). Baseline characteristics are shown in Table 1 . Compared with controls, patients were older, less often female, had a higher prevalence of cardiovascular risk factors, were more likely to be receiving treatments for risk factor modification, and had higher systolic blood pressures.

**Table 1:** Baseline characteristics of patients and controls

Characteristic	Controls ( *N* = 136)	Patients ( *N* = 115)	*P* value
Demographics			
Age	46.3 (11.7)	65.0 (10.7)	<0.0001
Female	107 (78.7%)	33 (28.7%)	<0.0001
CV risk factors			
Diabetes	2 (1.4%)	26 (22.6%)	<0.0001
Hypertension	10 (7.4%)	84 (73.0%)	<0.0001
Dyslipidemia	11 (8.1%)	101 (87.8%)	<0.0001
Current smoking	13 (9.6%)	28 (24.3%)	0.002
Past medical history			
CAD	0 (0%)	59 (51.3%)	–
PAD	0 (0%)	48 (41.7%)	–
Stroke/TIA	0 (0%)	15 (13.0%)	–
Medications			
Statins	11 (8.1%)	106 (92.2%)	<0.0001
Aspirin	2 (1.5%)	106 (92.2%)	<0.0001
ACE/ARB	5 (3.7%)	87 (75.7%)	<0.0001
CCB	0 (0.0%)	32 (27.8%)	<0.0001
Diuretics	3 (2.2%)	35 (30.4%)	<0.0001
Insulin	1 (0.7%)	6 (5.2%)	0.05
OHA	1 (0.7%)	17 (14.8%)	<0.0001
Beta blocker	2 (1.5%)	52 (45.2%)	<0.0001
Clinical measures			
Weight	76.6 (20.5)	83.1 (18.1)	0.0004
Height	166.3 (12.7)	171.0 (10.0)	0.0003
Systolic BP	122.8 (14.3)	135.5 (19.8)	<0.0001
Diastolic BP	73.9 (11.8)	76.1 (9.7)	0.11

Abbreviations: ACE, angiotensin converting enzyme inhibitor; ARB, angiotensin receptor blocker; BP, blood pressure; CAD, coronary artery disease; CCB, calcium channel blocker; CV, cardiovascular; N, number; OHA, oral hypoglycemic agent; PAD, peripheral artery disease; TIA, transient ischemic attack.

**Fig. 1:**
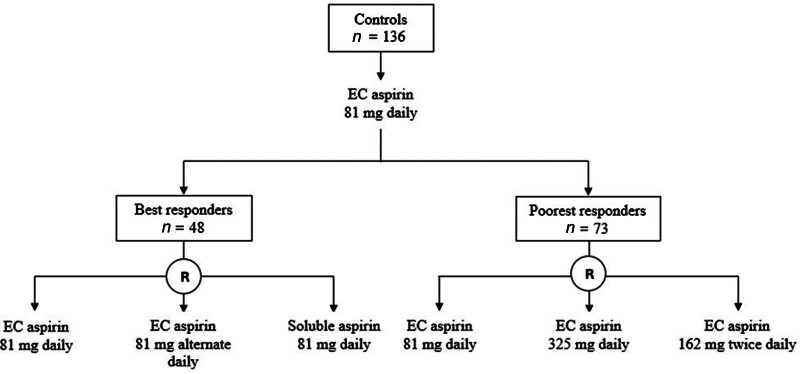
Study design and randomization schema for the control cohort. Controls ( *n* = 136) received enteric-coated (EC) aspirin 81 mg daily during phase 1. At the end of phase 1, 121 controls had evaluable response classification and entered phase 2 (best responders, *n* = 48; poorest responders, *n* = 73); 15 controls did not enter phase 2 (withdrawal or incomplete phase 1 assessment). In phase 2, best responders were randomized (R) to EC aspirin 81 mg daily, EC aspirin 81 mg alternate daily, or soluble aspirin 81 mg daily. Poorest responders were randomized to EC aspirin 81 mg daily, 325 mg daily, or EC aspirin 162 mg twice daily. Alternate daily indicates alternate daily dosing.

**Fig. 2:**
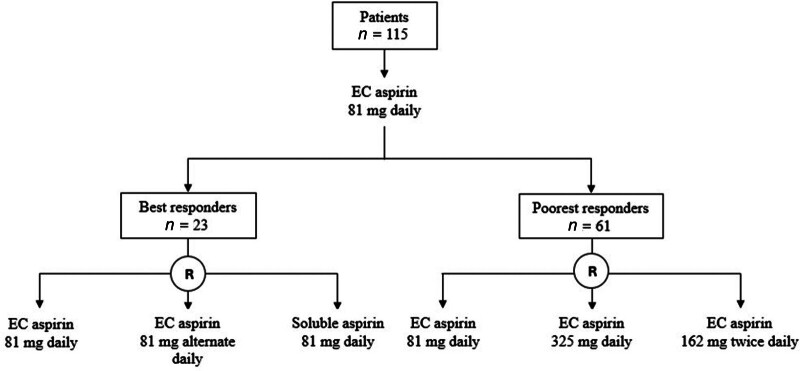
Study design and randomization schema for the patient cohort. Patients with established cardiovascular disease ( *n* = 115) received enteric-coated (EC) aspirin 81 mg daily during phase 1. At the end of phase 1, 84 patients had evaluable response classification and entered phase 2 (best responders, *n* = 23; poorest responders, *n* = 61); 31 patients did not enter phase 2 (withdrawal or incomplete phase 1 assessment). In phase 2, best responders were randomized (R) to EC aspirin 81 mg daily, EC aspirin 81 mg alternate daily, or soluble aspirin 81 mg daily. Poorest responders were randomized to EC aspirin 81 mg daily, EC aspirin 325 mg daily, or EC aspirin 162 mg twice daily. Alternate daily indicates alternate daily dosing.

Participant flow is shown in Figs. 1 and 2 .

### Phase 1: EC Aspirin 81 mg Once Daily

In controls, EC aspirin 81 mg once daily for 3 weeks suppressed aggregation induced by AA (median 86.0 [IQR 7.0] at baseline vs. 2.0 [IQR 1.0] at 3 weeks), ADP (80.0 [IQR 12.0] vs. 62.5 [IQR 12.0]), and collagen (84.0 [IQR 11.0] vs. 69.0 [IQR 22.0]), and reduced both sTXB2 (238.4 [IQR 206.5] vs. 2.7 [IQR 3.9] ng/mL) and uTXB2 (112.0 [IQR 78.0] vs. 39.0 [IQR 32.0] ng/mmol creatinine), all *p* < 0.0001 ( Table 2 ).

**Table 2:** Laboratory measures at baseline and after 3 weeks of EC aspirin 81 mg once daily in controls ( *n* = 136) and patients ( *n* = 115)

Laboratory measure	Controls ( *n* = 136) a		Patients ( *n* = 115)	
	Baseline median (IQR)	Week 3 median (IQR)	Week 3 median (IQR)	Controls vs. patients at 3 weeks *P* value
LT aggregation				
AA	86.0 (7.0)	2.0 (1.0)	2.0 (2.0)	0.20
ADP	80.0 (12.0)	62.5 (12.0)	61.0 (14.0)	0.32
Collagen	84.0 (11.0)	69.0 (22.0)	71.5 (20.0)	0.56
sTXB2 (ng/mL)	238.4 (206.5)	2.7 (3.9)	2.2 (3.3)	0.63
uTXB2 (ng/mmol cr)	112.0 (78.0)	39.0 (32.0)	46.5 (38.0)	0.004

Abbreviations: AA, arachidonic acid; ADP, adenosine diphosphate; cr, creatinine; IQR, interquartile range; LT, light transmission; mL, milliliter; mmol, millimole; *n* , number; ng, nanogram; sTXB2, serum thromboxane B2; uTXB2, urinary 11-dehydro-thromboxane B2.

a In controls, differences between baseline and week 3 were significant for all measures (all *p* < 0.0001).

After treatment with EC aspirin 81 mg once daily for 3 weeks, there were no significant differences between controls and patients for platelet aggregation or for sTXB2 (2.7 [IQR 3.9] vs. 2.2 [IQR 3.3] ng/mL; *p* = 0.63) but controls had significantly lower uTXB2 (39.0 [IQR 32.0] vs. 46.5 [IQR 38.0] ng/mmol creatinine; *p* = 0.004) ( Table 2 ).

### Phase 2: Best Responders

#### Controls

Aspirin formulation (EC vs. soluble) and dosing frequency (daily vs. alternate daily) had no consistent effect on AA- or ADP-induced aggregation or uTXB2 ( Table 3 , Panel A). An isolated difference in collagen-induced aggregation was observed in controls, but this was modest, not paralleled by AA-induced aggregation, and should be interpreted cautiously given multiple exploratory comparisons. Comparison between aspirin formulation also did not produce significantly different results for sTXB2, but alternate daily compared with daily aspirin dosing was associated with significantly higher sTXB2 for both EC aspirin 81 mg daily (8.1 [IQR 14.1] vs. 3.6 [IQR 4.7] ng/mL; *p* = 0.02) and soluble aspirin 81 mg daily (8.1 [IQR 14.1] vs. 2.0 [IQR 2.0] ng/mL; *p* = 0.02). Findings were similar when the two daily 81 mg arms (EC and soluble) were pooled ( Supplementary Table S1 , Panel A).

**Table 3:** Best responders: EC aspirin 81 mg daily versus EC aspirin 81 mg alternate daily versus soluble aspirin 81 mg daily

**Panel A: Controls**						
**Laboratory measure**	** EC aspirin 81 mg daily a** **Median (IQR)** **(** ***N*** ** = 18)**	**EC aspirin 81 mg alternate daily** **Median (IQR)** **(** ***N*** ** = 18)**	**Soluble aspirin 81 mg daily** **Median (IQR)** **(** ***N*** ** = 12)**	***P*** **value**		
				**Daily EC vs. alternate daily**	**Daily EC vs. soluble**	**Alternate daily vs. soluble**
LT aggregation						
AA	2.0 (1.0)	2.0 (1.0)	2.0 (2.0)	0.88	0.47	0.50
ADP	64.0 (10.0)	63.5 (9.0)	63.0 (16.5)	1.00	0.83	0.93
Collagen	70.5 (15.0)	77.0 (13.0)	66.5 (19.0)	0.10	0.29	0.02
sTXB2 (ng/mL)	3.6 (4.7)	8.1 (14.1)	2.0 (2.0)	0.02	0.34	0.02
uTXB2 (ng/mmol cr)	35.5 (24.0)	33.5 (23.0)	32.5 (19.5)	0.85	0.95	0.80
**Panel B: Patients**						
**Laboratory measure**	**EC aspirin 81 mg daily** **Median (IQR)** **(** ***N*** ** = 6)**	**EC aspirin 81 mg alternate daily** **Median (IQR)** **(** ***N*** ** = 5)**	**Soluble aspirin 81 mg daily** **Median (IQR)** **(** ***N*** ** = 12)**	***P*** **value**		
				**Daily EC vs. alternate daily**	**Daily EC vs. soluble**	**Alternate daily vs. soluble**
LT aggregation						
AA	2.0 (0.0)	1.0 (0.0)	2.0 (1.0)	0.33	0.26	0.07
ADP	56.5 (22.0)	64.0 (11.0)	55.5 (10.0)	0.85	0.61	0.15
Collagen	55.0 (23.0)	69.0 (5.0)	61.0 (16.5)	0.32	0.89	0.06
sTXB2 (ng/mL)	1.6 (2.8)	5.2 (2.2)	1.7 (2.5)	0.17	0.61	0.051
uTXB2 (ng/mmol cr)	28.0 (13.0)	47.0 (34.0)	32.0 (13.5)	0.17	0.85	0.21

Abbreviations: AA, arachidonic acid; ADP, adenosine diphosphate; cr, creatinine; IQR, interquartile range; LT, light transmission; mL, milliliter; mmol, millimole; *n* , number; ng, nanogram; sTXB2, serum thromboxane B2; uTXB2, urinary 11-dehydro-thromboxane B2.

a Missing data: serum thromboxane, *n* = 1.

#### Patients

Aspirin formulation (EC vs. soluble) and dosing frequency (daily vs. alternate daily) were not associated with significantly different results for platelet aggregation or uTXB2 ( Table 3 , Panel B). However, as with controls, sTXB2 was higher with alternate daily compared with daily dosing, and this difference was statistically significant when the two daily aspirin groups (EC and soluble) were pooled (5.2 [IQR 2.2] vs. 1.7 [IQR 2.7] ng/mL; *p* = 0.048) ( Supplementary Table S1 , Panel B).

### Phase 2: Poorest Responders

#### Controls

Modifying the aspirin dose and dosing frequency was not associated with statistically significant differences in platelet aggregation. However, EC aspirin 162 mg twice daily compared with 81 mg daily significantly reduced sTXB2 (0.5 [IQR 0.9] vs. 2.6 [IQR 7.6] ng/mL; *p* = 0.003) and uTXB2 (31.0 [IQR 17.0] vs. 48.0 [IQR 26.0] ng/mmol creatinine; *p* = 0.004), and compared with EC aspirin 325 mg daily significantly lowered sTXB2 (0.5 [IQR 0.9] vs. 1.5 [IQR 3.1] ng/mL; *p* = 0.03). EC aspirin 325 mg daily compared with EC aspirin 81 mg daily significantly lowered uTXB2 (35.0 [IQR 23.0] vs. 48.0 [IQR 26.0] ng/mmol creatinine; *p* = 0.04) ( Table 4 , Panel A). EC aspirin 162 mg twice daily produced the greatest thromboxane suppression, with intermediate suppression with EC aspirin 325 mg daily and least suppression with EC aspirin 81 mg daily (sTXB2: trend *p* = 0.0005; uTXB2: trend *p* = 0.01).

**Table 4:** Poorest responders: EC aspirin 81 mg daily versus EC aspirin 325 mg daily versus EC aspirin 162 mg twice daily

**Panel A: Controls**						
**Laboratory measure**	**EC aspirin 81 mg daily** **Median (IQR)** **(** ***N*** ** = 23)**	**EC aspirin 325 mg daily** **Median (IQR)** **(** ***N*** ** = 25)**	**EC aspirin 162 mg twice daily** **Median (IQR)** **(** ***N*** ** = 25)**	***P*** **value**		
				**Daily 81 mg vs. daily 325 mg**	**Daily 81 mg vs. twice daily 162 mg**	**Daily 325 mg vs. twice daily 162 mg**
LT aggregation						
AA	2.0 (2.0)	2.0 (1.0)	2.0 (0.0)	0.37	0.75	0.21
ADP	64.0 (9.0)	59.0 (12.0)	61.0 (10.0)	0.12	0.55	0.36
Collagen	69.0 (20.0)	65.0 (20.0)	67.0 (20.0)	0.21	0.48	0.37
sTXB2 (ng/mL)	2.6 (7.6) a	1.5 (3.1) a	0.5 (0.9) a	0.16	0.003	0.03
uTXB2 (ng/mmol cr)	48.0 (26.0) b	35.0 (23.0) b	31.0 (17.0) b	0.04	0.004	0.48
**Panel B: Patients**						
**Laboratory measure**		** EC aspirin 81 mg daily c** **Median (IQR)** **(** ***N*** ** = 22)**		** EC aspirin 325 mg daily d** **Median (IQR)** **(** ***N*** ** = 19)**	**EC aspirin 162 mg twice daily** **Median (IQR)** **(** ***N*** ** = 20)**	***P*** **value**		
						**Daily 81 mg vs. daily 325 mg**	**Daily 81 mg vs. twice daily 162 mg**	**Daily 325 mg vs. twice daily 162 mg**
LT aggregation						
AA	2.0 (1.0)	2.0 (2.0)	3.0 (1.5)	0.79	0.09	0.17
ADP	59.5 (13.0)	59.0 (14.0)	59.5 (8.5)	1.00	0.76	0.76
Collagen	64.5 (20.0)	59.0 (36.0)	55.5 (19.0)	0.31	0.19	0.92
sTXB2 (ng/mL)	1.6 (2.6) e	1.1 (0.8) e	0.8 (0.8) e	0.19	0.03	0.37
uTXB2 (ng/mmol cr)	62.5 (41.0) f	51.5 (48.0) f	42.0 (21.0) f	0.83	0.01	0.06

Abbreviations: AA, arachidonic acid; ADP, adenosine diphosphate; cr, creatinine; IQR, interquartile range; LT, light transmission; mL, milliliter; mmol, millimole; *n* , number; ng, nanogram; sTXB2, serum thromboxane B2; uTXB2, urinary 11-dehydro-thromboxane B2.

a Trend *p* = 0.0005.

b Trend *p* = 0.01.

c Missing data: serum thromboxane, *n* = 3; urinary thromboxane, *n* = 1.

d Missing data: serum thromboxane, *n* = 2; urinary thromboxane, *n* = 1.

e Trend *P* = 0.03.

f Trend *P* = 0.008.

#### Patients

Modifying the aspirin dose and dosing frequency was not associated with statistically significant differences in platelet aggregation. However, like controls, EC aspirin 162 mg twice daily compared with 81 mg daily significantly reduced sTXB2 (0.8 [IQR 0.8] vs. 1.6 [IQR 2.6] ng/mL; *p* = 0.03) and uTXB2 (42.0 [IQR 21.0] vs. 62.5 [IQR 41.0] ng/mmol creatinine, *p* = 0.01) ( Table 4 , Panel B). There were no significant differences between EC aspirin 162 mg twice daily and 325 mg daily, or between EC aspirin 325 mg daily and 81 mg daily. EC aspirin 162 mg twice daily produced the greatest thromboxane suppression, with intermediate suppression with EC aspirin 325 mg daily and the least suppression with EC aspirin 81 mg daily (sTXB2: trend *p* = 0.03; uTXB2: trend *p* = 0.008).

## Discussion

In this two-phase pharmacodynamic study, EC aspirin 81 mg once daily produced near-complete inhibition of platelet COX-1 activity as assessed by AA-induced aggregation and serum thromboxane B2 (sTXB2) in both controls and patients with established cardiovascular disease. Despite this uniform ex vivo suppression, urinary 11-dehydro-thromboxane B2 (uTXB2) remained incompletely suppressed and was higher in patients than controls, suggesting persistent in vivo thromboxane generation not detected by routine aggregation testing.

Alternate daily dosing attenuated biochemical suppression and, among the best responders, was associated with higher sTXB2, whereas in the poorest responders, aspirin dose intensification, particularly by shortening the dosing interval (162 mg twice daily) and less so by using a higher dose (325 mg daily), achieved greater suppression of serum and urinary thromboxane without materially affecting aggregation.

Several mechanisms might explain incomplete thromboxane suppression despite apparent consistency in aggregation suppression. Following aspirin ingestion, COX-1 in most circulating platelets is irreversibly acetylated, fully suppressing the platelet aggregation response to AA, even if a small fraction of uninhibited platelets released from the bone marrow during the 24-hour aspirin dosing interval can generate thromboxane. Accordingly, incomplete suppression of sTXB2 may reflect variable aspirin bioavailability, nonadherence, or drug interactions. Another important potential mechanism is accelerated platelet turnover in high-turnover states such as the postoperative period, systemic inflammation, and myeloproliferative neoplasms. In such settings, shortening the dosing interval can improve biochemical suppression by acetylating newly released platelets and narrowing the recovery window between doses. Our data support this since twice-daily dosing suppressed sTXB2 more completely than dose escalation alone, a pattern also reported in postoperative, myeloproliferative, diabetic, acute coronary syndrome, and vascular disease populations. 11 12 13 14 15 16 17 18 19 20

The dissociation between ex vivo aggregation and thromboxane biomarkers has been described in the literature on aspirin “non-responsiveness” (also referred to as aspirin “resistance”). In addition to the prognostic associations of uTXB2 reported by our group, 4 5 others have shown that EC aspirin may produce delayed or erratic absorption in some settings. 6 7 We have also shown that major orthopedic surgery and coronary artery bypass grafting are associated with increased thromboxane generation despite once-daily aspirin, and randomized studies have demonstrated improved suppression of thromboxane with more frequent aspirin dosing after cardiac surgery. 11 12 13

Taken together, these data suggest that strategies aimed at optimizing the dosing interval may be most relevant when thromboxane generation is driven by increased platelet turnover and/or heightened inflammatory activation.

Importantly, neither previous studies nor ours were designed or powered to evaluate clinical outcomes. Therefore, although more frequent and higher aspirin dosing can further suppress thromboxane production compared with daily low-dose aspirin in high-turnover settings and in some cases may even improve aggregation suppression, whether this translates into fewer graft occlusions or ischemic events (and at what bleeding cost) remains unknown. The apparent paradox is that higher aspirin doses have not shown greater clinical benefit than low-dose regimens in chronic secondary prevention. 1 3 A plausible explanation is that low-dose aspirin already produces near-maximal irreversible platelet COX-1 inhibition in most patients with stable cardiovascular disease; residual uTXB2 may reflect thromboxane generation from extra-platelet sources (including COX-2-dependent inflammatory pathways), or variable adherence/absorption, which is not reliably overcome by dose escalation. 5 6 7 8 In addition, higher doses may not only increase the risk of gastrointestinal bleeding but may also suppress vascular-protective prostacyclin production, thereby potentially offsetting any incremental antithrombotic effect. 1 3

Strengths of this study include standardized aspirin treatment, parallel evaluation in controls and patients, and complementary assessment of platelet COX-1 activity (sTXB2) and integrated in vivo thromboxane generation (uTXB2). Limitations include the open-label design, modest sample sizes in some subgroups, and reliance on pharmacodynamic rather than clinical endpoints. Reasons for withdrawal or incomplete phase participation, including aspirin intolerance, were not systematically recorded; therefore, the study cannot assess comparative tolerability or safety of the tested aspirin regimens. Clinically, the findings caution against using ex vivo aggregation alone to infer adequate aspirin effect and support evaluating more frequent aspirin dosing in select high-risk or high-turnover settings.

In conclusion, low-dose aspirin uniformly suppresses ex vivo AA-induced platelet aggregation and profoundly inhibits serum thromboxane generation, but urinary thromboxane metabolites remain incompletely suppressed, especially in patients with established cardiovascular disease. Alternate daily dosing attenuates biochemical suppression, while twice daily aspirin improves suppression in poorest responders, supporting a role for dosing frequency in optimizing biochemical thromboxane inhibition.

## Notes

**What is Known About this Topic?**

- Low-dose aspirin reliably inhibits platelet COX-1 ex vivo, but biochemical thromboxane suppression in vivo is variable.
- Variability in thromboxane suppression has been linked to formulation effects, delayed absorption, and missed dosing, and predicts future cardiovascular risk.

**What Does this Paper Add?**

- Enteric-coated aspirin 81 mg once daily produced uniform suppression of AA-induced aggregation and serum thromboxane in both controls and patients with cardiovascular disease, but urinary thromboxane remained higher in patients.
- In the best responders, soluble and enteric-coated aspirin 81 mg daily performed similarly, whereas alternate daily dosing measurably worsened thromboxane suppression.
- In the poorest responders, enteric-coated aspirin 162 mg twice daily produced the greatest suppression of both sTXB2 and uTXB2, with intermediate suppression with enteric-coated aspirin 325 mg daily and the least with enteric-coated aspirin 81 mg daily.
- These findings support dosing interval as an effective approach to optimizing biochemical thromboxane suppression and provide a rationale for outcome trials in settings of increased thromboxane generation (e.g., high platelet turnover).
