# Supplementary material for: Aspirin Dosing Frequency and Dose Influence Thromboxane Suppression Despite Uniform Inhibition of Arachidonic Acid-induced Platelet Aggregation
Source: TH Open. 2026 Jun 12;10:a28852306. doi: 10.1055/a-2885-2306 (PMC13289645; doi:10.1055/a-2885-2306)
Supplement: Supplementary file 1 — Supplementary Material [file 10-1055-a-2885-2306_28948992.pdf]

Table S1. Best responders: Aspirin 81 mg daily (pooled EC and soluble) vs EC aspirin 81 mg alternate daily

Panel A. Controls

**Laboratory measure**	Aspirin 81 mg daily (pooled EC + soluble) (N=30)	Aspirin 81 mg alternate daily (EC) (N=18)	P value
AA-induced aggregation (%)	2.0 (2.0)	2.0 (1.0)	0.82
ADP-induced aggregation (%)	63.0 (11.0)	63.5 (9.0)	0.97
Collagen-induced aggregation (%)	70.5 (16.0)	77.0 (13.0)	0.02
Serum thromboxane B2 (ng/mL)	2.2 (3.7)	8.1 (14.1)	0.005
Urine 11-dehydro thromboxane B2 (ng/mmol creatinine)	34.0 (22.0)	33.5 (23.0)	0.79

Panel B. Patients

**Laboratory measure**	Aspirin 81 mg daily (pooled EC + soluble) (N=18)	Aspirin 81 mg alternate daily (EC) (N=5)	P value
AA-induced aggregation (%)	2.0 (0.0)	1.0 (0.0)	0.08
ADP-induced aggregation (%)	56.0 (8.0)	64.0 (11.0)	0.26
Collagen-induced aggregation (%)	57.5 (15.0)	69.0 (5.0)	0.08
Serum thromboxane B2 (ng/mL)	1.7 (2.7)	5.2 (2.2)	0.048
Urine 11-dehydro thromboxane B2 (ng/mmol creatinine)	30.0 (11.0)	47.0 (34.0)	0.14

AA, arachidonic acid; ADP, adenosine diphosphate; cr, creatinine; IQR, interquartile range; LT, light transmission; mmol, millimole; mL, milliliter; n, number; ng, nanogram; sTXB2, serum thromboxane B2; uTXB2, urinary thromboxane B2
